# The perceived role fit of women and men academics: evidence from the social sports sciences

**DOI:** 10.3389/fpsyg.2023.1239944

**Published:** 2023-11-20

**Authors:** Lara Lesch, Katrin Scharfenkamp, Pamela Wicker

**Affiliations:** Department of Sports Science, Bielefeld University, Bielefeld, Germany

**Keywords:** gender stereotypes, social roles, role congruity, role attributes, sport management/economics/sociology, higher education

## Abstract

**Introduction:**

The underrepresentation of women in academia is often explained by the presence of gender stereotypes and the perception that women fit the role of an academic to a lesser extent. Based on social role theory and role congruity theory, this study investigates and estimates the perceived role fit of women and men academics in the social sports sciences.

**Methods:**

Data were collected with a quantitative online survey. The sample (*n* = 792) includes individuals who study or work in sports economics, sport management, or sport sociology (referred to as social sports sciences). The questionnaire included items that reflect attributes of an ideal-typical academic as well as women and men academics in four dimensions, i.e., leadership, research methods, media visibility, and research topics. In the first step, these items were used to estimate a total role fit index for both women and men academics, as well as indices for all dimensions. In a second step, regression analyses were used to examine how respondents' individual characteristics (e.g., discipline, career stage, gender, presence of role models) are related to their perceived role fit indices and the differences in the perceived role fit.

**Results and discussion:**

The role fit index ranges from 0 to 1, and women have a higher total role fit than men (0.77 vs. 0.75). The results suggest that women in the social sports sciences are perceived as a better fit for the role of an academic. In contrast to role congruity theory, women's leadership fit is higher than men's fit in this dimension (0.79 vs. 0.72). Regarding the associations of individual characteristics, professors seem to perceive a lower role fit for both genders than students. Furthermore, the difference between the perceived role fit of men and women is smaller for women respondents. Having a woman role model leads to a higher fit of women academics in the leadership dimension.

## 1 Introduction

In 2019, only 29.3% of the world's researchers were women (UNESCO Institute for Statistics, 2019). However, the representation of women in academia highly depends on the career stage and differs between disciplines: even though more women than men are enrolled in undergraduate and postgraduate programs in the European Union (Eurostat, 2020), women occupy only 26.2% of professor positions (European Commission, 2021). Furthermore, women are underrepresented in typically men-dominated disciplines like engineering and technology. They are more likely to work in the social and health sciences (European Commission, 2021).

The underrepresentation of women academics in the social sports sciences is a global phenomenon. For example, only 24.5% of German sport professors are women (Federal Statistical Office, 2022). This low share is not surprising given that sport is generally perceived as a masculine field (Burton, 2015). The social sports sciences include sports economics, sport management, and sport sociology. No study has investigated women's representation in sports economics. However, for general economics, only 15% of full professors in the United States (US) are women, underlining that economics is a men-dominated discipline (Lundberg and Stearns, 2019). In sport management faculties in the US, 45.9% of assistant professors and 37.7% of full professors are women (Sailofsky et al., 2023), indicating that women's representation seems to decrease with hierarchical higher positions. No study indicates the number or share of women in sport sociology. However, in the superordinate discipline of sociology, 42.7% of full professors in the US are women (Casad et al., 2022).

One common explanation for women's underrepresentation in academia is based on the perception that women do not fit the role of an academic (Carli et al., 2016). This lack-of-fit between the job role and the social gender role of women is the result of a comparison between the perceived attributes of women and men (gender attributes; Eagly, 1987) and perceived relevant attributes for a job (Heilman, 2012). Role fit can be defined as “the complex integration of characteristics of the person (their abilities, interests, goals, and values) and of the situation (what the career is, where it is done, how work is structured, and who tends to do the work)” (Schmader, 2023, p. 223). Generally speaking, “science is male” (Symth and Nosek, 2015, p. 1). Intellectual knowledge is more attributed to men than women (Bailey et al., 2019). The gendered perception of jobs in general and academia in particular can be explained through prevalent and historically grown gender stereotypes (e.g., Ertl et al., 2017; Branchefsky and Park, 2018; van Veelen and Derks, 2022a). These stereotypes shape people's perception of women's and men's role fit and if an academic position in a certain discipline is appropriate for women or men (Carli et al., 2016).

However, different levels of gender-science stereotypes exist in different disciplines (Leslie et al., 2015). Previous studies mostly investigated women's presence and gender stereotypes in science, technology, engineering, and mathematics (STEM), in which women are historically underrepresented (e.g., Ertl et al., 2017; McGuire et al., 2022). The focus on STEM disciplines results in a research gap for the social sciences (Johnson et al., 2022). While women are overrepresented in some disciplines in the social sciences, they remain minorities in others (Casad et al., 2022). For example, more women than men work in psychology and sociology in the US, while most academic faculty members in political sciences and economics are men (Casad et al., 2022).

Given this heterogeneous picture, it is important to investigate the attributes which are associated with an ideal-typical academic in the social sports sciences and how women and men academics fit this academic role. The first purpose of this study is to estimate the perceived role fit of women and men academics in the social sports sciences. In a study conducted by Carli et al. (2016) in the US, undergraduate students perceived a greater fit between a successful scientist in STEM disciplines and men attributes. However, the perceived role fit has not been empirically calculated, and the social sciences were not the focus of the research. The second purpose is to examine how individual characteristics of people who work or study in the social sports sciences are related to their perceived role fit of women and men academics in the three disciplines. The third purpose is to investigate which individual characteristics might explain a different perception of women and men academics in the social sports sciences. While previous research has investigated gender stereotypes and how their perception differs between gender and career stage in society (e.g., Koenig, 2018; Haines et al., 2019) and in STEM disciplines (e.g., Bailey et al., 2019), the social sciences were not in the focus of research (Johnson et al., 2022).

Specifically, this study addresses the following three research questions: (1) What is the perceived role fit of women and men academics in the social sports sciences? (2) Which individual characteristics are related to the perceived role fit? And (3) Which individual characteristics are related to the difference in perceived role fit for women and men academics? Theoretically grounded in social role theory and role congruity theory, the research questions are analyzed using a dataset gathered from a quantitative online survey targeted at people at different academic career stages in the social sports sciences. Investigating and understanding the role fit of women (and men) academics is important to recruit and retain more women in the social sports sciences since women are already minorities in the sports sciences (e.g., Federal Statistical Office, 2022). Gender stereotypes were found to have a negative impact on women's science career aspirations (Cundiff et al., 2013). Thus, gaining knowledge about the perception of role fit might help to actively tackle gender stereotypes and implement measures to reduce these stereotypes within the social sports sciences, making the disciplines more attractive for women.

## 2 Theoretical framework and literature review

### 2.1 Social role theory and role congruity theory

According to social role theory (Eagly, 1987; Eagly et al., 2000), gender stereotypes are closely linked to traditional roles which women and men should fulfill in society. These societal roles result in gendered expectations about appropriate behavior for women and men (Eagly, 1987). Thus, gender stereotypes reflect which attributes women and men should have (Prentice and Carranza, 2002). The ascribed gender roles are not only reflective of gendered attributes but also qualities of women and men and desired behaviors (Eagly, 1987). Historically, men participated in the labor force and economically cared for their families. At the same time, women focused on homemaker and childcare work (Eagly et al., 2000). Therefore, women's social role is associated with communal attributes such as being warm, caring, sensitive, and compassionate. In contrast, men's social role includes agentic attributes, such as being dominant, analytical, self-sufficient and having leadership abilities (Eagly and Karau, 2002; Prentice and Carranza, 2002).

Gender stereotypes do not only include beliefs about attributes and behaviors but also about cognitive skills and perceived adequate occupations of women and men (Diekman and Eagly, 2000). Accordingly, the segregation in social roles leads to power and opportunity inequalities since women and men learn which skills are relevant to their social gender roles, resulting in different treatment and the perception of desirable careers (Meussen et al., 2022). If women and men work in jobs which are in line with their gender roles, society perceives them as “successful and productive members” who fulfill the gendered role expectations (Clow and Ricciardelli, 2011, p. 198). When women and men enter a work environment that is dominated by the other gender, the assumed lack-of-fit between the social gender role and the job position results in conflicts and the perception of inadequacy (Heilman, 2012). This notion is reflected in role congruity theory (Eagly and Karau, 2002), assuming that prejudices become relevant when the social role is not congruent with the attributes and requirements of a certain job position. Especially jobs which require leadership skills violate the social role and related attributes of women (Prentice and Carranza, 2002). Accordingly, women in such positions are negatively evaluated because they neither fit the role of the position nor the women's gender role (Eagly and Karau, 2002).

Even though men are still more likely than women to have higher status jobs (Eagly and Wood, 2011), some studies indicate that women's increased participation in the labor force has led to a convergence of roles (Diekman and Eagly, 2000). Women are perceived as gaining in power (Diekman et al., 2004). They are also associated with masculine attributes if they work in men-dominated fields or positions (Diekman and Eagly, 2000; Sendén et al., 2019). However, other studies did not find any changes in gender stereotypes or gender roles (Haines et al., 2019).

### 2.2 Role fit and gender stereotypes of women and men academics

In line with role congruity theory (Eagly and Karau, 2002), the stereotypes which are associated with a successful academic are agentic (i.e., favor attributes ascribed to men), while communal (i.e., attributes ascribed to women) attributes were perceived as less important (van Veelen and Derks, 2022b). People who work in academia are expected to be men and have math knowledge (Nosek and Smyth, 2011), resulting in the stereotypical view that “women do not have what it takes” (van Veelen and Derks, 2022b, p. 750) to work in academia. Disciplines which are men-dominated are related to stronger and more negative stereotypes about women's fit to the discipline, and women were found to be less accepted in these disciplines (Branchefsky and Park, 2018). Accordingly, the perceived role fit might be related to the presence of women within a discipline since a higher share of women academics leads to a stronger association between women's attributes and those of a successful academic (Carli et al., 2016). The social sports sciences include disciplines which are more closely related to men attributes (sport economics), but also disciplines in which women attributes are valued (sport sociology). Therefore, it is important to investigate attributes of academics in the social sports sciences and estimate the perceived role fit. In addition to the work of van Veelen and Derks (2022b), no study has yet calculated a role index for women and men academics.

The investigation of perceived role attributes and the calculation of role fit of women and men academics is particularly valuable because some studies have produced contrary results on the perception of women in science. While competence is stereotypically perceived as a men attribute (Prentice and Carranza, 2002), women in both academia and leadership positions were rated similar or higher in competence than men (Bye et al., 2022). Similarly, women lecturers were not perceived as different to their men colleagues by students (Renström et al., 2021).

Research in sports economics is focused on the economic value, nature, and impact of professional sports systems/leagues, team performance, and the sports labor market (Downward et al., 2019). The large majority of published studies in sports economics use quantitative methods and statistical analyses to investigate men sports (Mondello and Pedersen, 2003). Sport management investigates topics related to the business of sports, including recreational and community sports, communication, ethics, governance, gender and inclusion (Pitts et al., 2014). Applied methods are both quantitative and qualitative and include (among others) data collection with surveys or interviews (Damon et al., 2020). In sport sociology, typical research topics are related to social and cultural aspects of sports, sport participation and health, gender aspects and behavior of individuals and groups within sports (Wicker et al., 2022). Thus, sport sociology is focused on people and behavior (McPherson, 1975), and research is conducted both quantitatively and qualitatively.

For the purpose of this study, the perception of attributes of women and men academics in the social sports sciences are considered in the four dimensions leadership, research methods, research topics, and media visibility. These dimensions are relevant for the work as an academic. While leadership and media visibility might be important for academics across all disciplines, attributes which are required or perceived in the dimensions of research methods and research topics are discipline-specific.

Starting with the leadership dimension, the stereotype of women being less competent than men was found to have an impact on hiring practices in academia (e.g., Moss-Racusin et al., 2012). In addition to competence, leadership is associated with authority and power, which are attributed to the social role of men (Prentice and Carranza, 2002). Women and men are not only perceived as having different leadership attributes but also related to different leadership styles. Women's leadership style is often more democratic and participative, while the style of men tends to be directive and top-down (Eagly and Johnson, 1990; Eagly and Johannesen-Schmidt, 2001). The “think manager—think male” stereotype (O'Connor, 2014, p. 109) might also exist in the social sports sciences, and women might have problems fitting in any of their roles: if women behave like women, they cannot fit the role and expectations of a leader (Forsyth and Nye, 2008). However, if women behave like leaders, they do not fit into their socially constructed gender roles (O'Connor, 2014). A recent study by Tremmel and Wahl (2023) suggested that the mismatch between women's social roles and the role of a leader might have become smaller since women leaders were rated more positively than men leaders. Furthermore, initiatives in politics and the economy (e.g., gender quotas) might have an impact on the perception of gender stereotypes (De Paola et al., 2010; Mölders et al., 2018). Giving this mixed evidence, leadership and how attributes are associated with women and men academics need further investigation.

Turning to research methods, women face the stereotype of being less mathematically talented (Cadinu et al., 2005) and lacking technical and analytical skills (Calanca et al., 2019). Women are stereotyped as being verbally more fluent (Mòe et al., 2021) and more competent in language arts (Plante et al., 2019). In academia, women are minorities in disciplines in which quantitative research is performed (Bettinger and Long, 2005), e.g., in sports economics (Mondello and Pedersen, 2003). Based on these findings, attributes related to research methods in academia might be perceived as stereotypically masculine, potentially resulting in a better role fit of men academics in the social sports sciences.

Gender stereotypes might impact not only the presence of women but also their interest in research topics. Gender stereotypes already have an impact on children's study interests (Steffens et al., 2010). For academic field choices, Su et al. (2009) suggested that women tend to select majors in which they work with people, while men choose majors in which they work with things. This preference is reflected in research topics: women are more present in research related to gender, health, or education. Contrary, they are less present in research related to finances, econometrics, or statistics (Thewall et al., 2019; Conde-Ruiz et al., 2022). Accordingly, it might be possible that research in the social sports sciences related to inclusion and gender diversity topics is more attributed to women academics, while statistics-related topics like performance and competition might be perceived as a stereotypically men domain.

Finally, multiple studies have investigated the visibility of women and men academics in the media. Electronic media were found to be the main source of career information for students aged 16–21 years (Hassan et al., 2022). In newspapers, women scientists' visibility has increased over the past decade but is limited to regional news sections (Eizmendi-Iraola and Peña-Fernández, 2023). On television, men are more often invited as scientific experts in talk shows (Hetsroni and Lowenstein, 2014). Women academics are also less visible in academic journals in the social sports sciences: only 10.6% of authors who published in the Journal of Sports Economics between 2000 and 2019 were women (Gomez-Gonzalez et al., 2022), and women make up 36% of authors in the Journal of Sport Management between 1984 and 2012 (Pitts et al., 2014). In the European Journal for Sport and Society, the share of women authors was 27.7% for publications between 2004 and 2020 (Wicker et al., 2022).

### 2.3 Individual characteristics and perceived role fit: literature review and hypotheses

Individual characteristics might be related to the perceived role fit of women and men academics. These characteristics include the academic discipline, career stage, gender, the presence or absence of women and men role models, and the country individuals live in. First, the academic discipline, i.e., if a person works or studies in sport economics, management, or sociology, might be related to the perception of attributes and role fit. The perception of gender-science stereotypes is stronger in men-dominated disciplines and weaker in disciplines with a higher women representation (Symth and Nosek, 2015; Branchefsky and Park, 2018). Gendered beliefs that men are naturally more talented and have the necessary discipline-specific skills are more present in those men-dominated disciplines (Leslie et al., 2015). In the social sports sciences, sport economics and sport management are men-dominated disciplines, indicated by the low share of women coauthors in sports economics and sport management journals (Pitts et al., 2014; Gomez-Gonzalez et al., 2022). Previous studies reported an unequal gender distribution in sport management programs and faculties (e.g., Jones et al., 2008; Sailofsky et al., 2023). Furthermore, skills which are stereotypically attributed to men, e.g., mathematical, analytical, and statistical skills (Ginther and Kahn, 2004), are valued in economics. Even though women are also minorities in an academic sport sociology journal (Wicker et al., 2022), the culture within the discipline might be more valuable toward women academics. Women are nearly equally represented in the subordinate discipline of sociology (Casad et al., 2022). Thus, sport sociology seems to better fit women's tendency to study people and not things (Su et al., 2009). These findings lead to the first hypothesis:

- **Hypothesis 1a:** Individuals in sport sociology perceive a higher role fit for women academics than individuals in sports economics and sport management.
- **Hypothesis 1b:** Individuals in sports economics and sport management perceive a higher role fit for men academics than individuals in sport sociology.

Secondly, the perceived role fit might be related to the career stage of the observing person. Women in earlier career stages were found to perceive a higher lack-of-fit between a successful academic and themselves. In contrast, both women and men full professors perceived a higher fit (van Veelen and Derks, 2022b). Women undergraduate students think that men are more likely to pursue successful careers than women, indicating that those women students tend to attribute success at a later career stage to the social role of men (Ollrogge et al., 2022). In a qualitative interview study with professors, agentic (i.e., masculine) attributes were perceived as more important for individuals in the pre-tenure stages. In contrast, communal (i.e., feminine) attributes were related to later academic career stages (Rehbock et al., 2021). These findings result in the second hypothesis:

- **Hypothesis 2a:** Individuals in early career stages perceive a higher role fit for men academics.
- **Hypothesis 2b:** Individuals in early career stages perceive a lower role fit for women academics.

Thirdly, women and men might perceive gender role attributes differently. Men were found to perceive less congruity between women and work as a scientist (Carli et al., 2016). Men academics have stronger gender-science stereotypes (Symth and Nosek, 2015) and perceive women as less qualified for leadership positions than men academics (Eagly and Karau, 2002). Similarly, men rated women as less agentic than men, e.g., having less leadership competence and assertiveness, in a study conducted by Haines et al. (2019). Bye et al. (2022) found that women perceive women academics as more competent than men academics. Furthermore, both women and men perceive that traditional gender roles change. However, compared to men, women rate it more positively that women gain in power (Diekman et al., 2004). Therefore, it is likely that women perceive a higher role fit for women academics than men. The third pair of hypotheses reflect these findings:

- **Hypothesis 3a:** Women perceive a higher role fit for women academics than men.
- **Hypothesis 3b**: Men perceive a higher role fit for men academics and a lower role fit for women academics.

Fourthly, the presence or absence of role models in the social sports sciences might be associated with the perceived role fit of women and men academics. Studies suggest that same-gender role models can help to increase the perceived role fit because role models underline the appropriateness of their behavior for their gender (Schunk and Usher, 2019). Women role models in academia were described as especially important for women in men-dominated disciplines because these role models represent the possibility to have career success, even though the perceived role incongruity might be higher in these disciplines (Lockwood, 2006). Additionally, these role models positively influence women's perception of women stereotypes (Dasgupta and Asgari, 2004; Olsson and Martiny, 2018). Given the relationship between role model gender and gender stereotypes, it might be possible that people perceive a higher or lower role fit for women and men academics depending on the gender of their role model. Therefore, the fourth set of hypotheses is formulated as follows:

- **Hypothesis 4a:** Individuals with a woman role model perceive a higher role fit for women academics.
- **Hypothesis 4b**: Individuals with a man role model perceive a higher role fit for men academics.

Fifthly, people from different countries might have different levels of gender stereotypes and assignment of role attributes based on different levels of gender equality in society, businesses, and politics in the countries. Gender-science stereotypes were found to be stronger in countries with a higher general gender equality gap (Mòe et al., 2021). Furthermore, a stronger lack-of-fit between the social role of women and an incongruent job is more negatively evaluated in countries with conservative political ideologies (Hoyt, 2012). According to the global gender gap report (World Economic Forum, 2022), the gender gap is an index which is based on the level of gender equality in economic participation, educational attainment, health and survival, and political empowerment. In terms of regions, the gender gap is closed by 76.9% in North America, 76.6% in Europe, and 69% in East Asia and the Pacific (World Economic Forum, 2022). The US and Canada are ranked higher for the dimensions of economic participation (22 and 43, respectively) and educational attainment (1 and 51, respectively) than Germany (75 and 81) and Austria (81 and 61). These aspects lead to the last hypothesis:

- **Hypothesis 5a:** Individuals who study or work in the US or Canada perceive a higher role fit for women academics.
- **Hypothesis 5b**: Individuals who study or work in Germany or Austria perceive a higher role fit for men academics.

## 3 Method

### 3.1 Procedure and participants

Data were gathered with a quantitative online survey from June 2022 to January 2023. The survey was programmed on the platform www.soscisurvey.de. It was targeted at students (undergraduate, postgraduate, PhD), post-doc researchers, and professors in sports economics, management, and sociology. The link to the survey was distributed in two ways: firstly, the survey was promoted after seven conferences in the three disciplines. More specifically, conference participants were requested to participate in the survey via email and Twitter. Secondly, academics in the three disciplines were contacted via email and were asked to participate themselves and share the link with their students and faculty colleagues. These emails were sent to more than 300 academics in Australia, Austria, Canada, Germany, the United Kingdom (UK), and the US.

The final sample size includes *n* = 792 participants. Overall, 38.8% of respondents studied or worked in the field of sports economics. Half of the respondents (50.3%) studied or worked in sport sociology. In contrast, two-thirds of respondents' study program or work was related to sport management. Respondents' ages ranged between 18 and 80 years, with an average age of 27 years. Most respondents were undergraduate or graduate students (65%), while 15.4% were PhD students and 14.1% were professors. More than half of the respondents (59.1%) were men, and more respondents had a man professor as a role model (35.9%) than a woman professor as a role model (32.4%). The vast majority of respondents studied or worked in Germany (60.6%), followed by the US (17.8%) and Canada (7.8%).

### 3.2 Questionnaire

The questionnaire was designed as part of a larger research project. At the beginning of the questionnaire, respondents were informed about the purpose of the study, data treatment and data publication, ethical conduct, and voluntary participation. Table 1 provides an overview of all variables used for the present empirical analysis.

**Table 1:** Overview of variables and summary statistics (*n* = 792).

**Variable**	**Description and codes**	**Mean**	**SD**	**Min**	**Max**
Fit W_Total	Total role fit index for women academics (0 = no fit; 1 = perfect fit)	0.77	0.11	0.18	1
Fit M_Total	Total role fit index for men academics (0–1)	0.75	0.11	0.33	1
Fit Diff_Total	Absolute difference between Fit M_Total and Fit W_Total	−0.01	0.10	−0.38	0.65
Fit W_Leader	Leadership fit index for women academics (0–1)	0.79	0.15	0.12	1
Fit M_Leader	Leadership fit index for men academics (0–1)	0.72	0.19	0	1
Fit W_Methods	Research methods fit index for women academics (0–1)	0.81	0.15	0	1
Fit M_Methods	Research methods fit index for men academics (0–1)	0.82	0.13	0.12	1
Fit W_Research	Research topics fit index for women academics (0–1)	0.82	0.15	0.13	1
Fit M_Research	Research topics fit index for men academics (0–1)	0.81	0.14	0.25	1
Fit W_Media	Media visibility fit index for women academics (0–1)	0.76	0.15	0.10	1
Fit M_Media	Media visibility fit index for men academics (0–1)	0.77	0.15	0.13	1
Economics	Sports economics is part of respondent's study/work (1 = yes)	0.388	—	0	1
Management	Sport management is part of respondent's study/work (1 = yes)	0.663	—	0	1
Sociology	Sport sociology is part of respondent's study/work (1 = yes)	0.503	—	0	1
Student	Respondent is a Bachelor or Master student (1 = yes)	0.650	—	0	1
PhD student	Respondent is a PhD student (1 = yes)	0.154	—	0	1
Post-doc	Respondent is a post–doc researcher (1 = yes)	0.054	—	0	1
Professor	Respondent is a professor (1 = yes)	0.141	—	0	1
Woman	Respondent is a woman (1 = yes)	0.409	—	0	1
Woman_Prof_RM	Respondent has a woman professor as role model (1 = yes)	0.324	—	0	1
Man_Prof_RM	Respondent has a man professor as role model (1 = yes)	0.359	—	0	1
Germany	Respondent studies/works at a university in Germany (1 = yes)	0.606	—	0	1
US	Respondent studies/works at a university in the USA (1 = yes)	0.178	—	0	1
Canada	Respondent studies/works at a university in Canada (1 = yes)	0.078	—	0	1
Australia	Respondent studies/works at a university in Australia (1 = yes)	0.033	—	0	1
Austria	Respondent studies/works at a university in Austria (1 = yes)	0.030	—	0	1
UK	Respondent studies/works at a university in UK (1 = yes)	0.029	—	0	1
Other_Country	Respondent studies/works at a university in one of 20 other countries that are represented by < 1% in the sample (e.g., Belgium, Sweden, Slovakia, New Zealand, 1 = yes)	0.045	—	0	1
Science attitude	Science attitude index (1 = low science attitude; 5 = strong science attitude)	3.23	0.034	1	5

The perception of role attributes of an ideal-typical academic, as well as the perception of women and men academics in the social sports sciences, was assessed with three identical scales. Each item was measured with a 5-point Likert scale from 1 (strongly disagree) to 5 (strongly agree). The three scales were presented to participants in a randomized order. Such explicit measures are traditionally used for investigating attributes of women and men and gender stereotypes by asking participants to rate how likely or desirable certain attributes are for persons in general, a man, or a woman (Haines et al., 2019; Tremmel and Wahl, 2023). The overall scale included 16 items, with four items in each dimension. According to Haines et al. (2019), such a multi-dimensional approach is beneficial to examine how attributes are assigned in different areas of interest. The items were created by grouping attributes, which have been investigated in previous studies (e.g., Sczesny et al., 2004; Cadinu et al., 2005; Calanca et al., 2019; Thewall et al., 2019) in the four dimensions of leadership, research methods, media visibility, and research topics. Table 2 shows all dimensions and items using the example of the scale for ideal-typical academics in the social sports sciences.

**Table 2:** Dimensions and items of the role fit scale (1 = strongly disagree; 5 = strongly agree; *n* = 792).

***“Academics in sport management/economics/sociology should have the following attributes:”***	**Mean**	**Cronbach's α**
**Leadership style**		
Authoritarian	2.69	0.704
Power-seeking	2.04	
Cooperative^a^	4.57	
Solution-oriented in conflict situations^a^	4.50	
**Quantitative methods**		
Analytical	4.27	0.834
Statistically competent	4.05	
Good with numbers	3.82	
Able to handle large data sets	3.83	
**Research topics**		
Knowledgeable in the field of professional sport leagues	3.86	0.861
Knowledgeable in the field of community sport	3.93	
Knowledgeable in the field of sport performance and competition	3.89	
Knowledgeable in the field of inclusion and diversity in sport	3.98	
**Media visibility**		
Visible in the media	2.77	0.819
Visible on social media platforms by sharing scientific content	2.78	
Visible in scientific journals	3.54	
Visible as experts on television	2.58	
All items		0.755

a Reverse-coded items.

The four items for the leadership dimension are based on previous studies focused on gender stereotypes and task-oriented as well as person-oriented attributes of leaders (e.g., Sczesny, 2003; Sczesny et al., 2004) and studies on leadership styles (e.g., Eagly and Johnson, 1990). However, it must be noted that leadership was not measured directly but indirectly by using certain attributes of leaders as items in the scale. This approach is called “the trait approach” (Gregoire and Arendt, 2004, p. 395). It reflects the measurement of leadership with attributes that are considered important for a leader. Furthermore, two items reflected stereotypically feminine attributes and were reverse coded. Therefore, they were recoded for the analysis.

For research methods, four items were included reflecting findings on math and statistics stereotypes (e.g., Ginther and Kahn, 2004; Cadinu et al., 2005) and the gendered perception of quantitative research methods which require analytical skills (Bettinger and Long, 2005; Calanca et al., 2019).

The items for the dimension research topics were developed for the purpose of the questionnaire and reflect typical research areas in the social sports sciences but are based on findings on gender differences in research topics (Thewall et al., 2019).

Finally, the items for media visibility reflect the visibility in different media outputs, i.e., social media, scientific journals, and television, which are considered relevant for academics (Hetsroni and Lowenstein, 2014; Hassan et al., 2022; Wicker et al., 2022).

The overall scale with all 16 items and all four dimensions were tested for construct reliability with Cronbach's alpha (see Table 2). Cronbach's alpha ranges between 0 and 1 and is an indicator of the internal consistency of a construct (Spicer, 2005). In this study, the value for the overall scale is 0.755, while the values for the dimensions are 0.704 for leadership, 0.834 for research methods, 0.819 for media visibility, and 0.861 for research methods. These values indicate acceptable to strong construct reliability because they are all above the suggested threshold of 0.700 (Spicer, 2005).

Respondents were also asked to answer questions related to their individual characteristics, which were considered as related to the perception of gender stereotypes in previous literature (e.g., Carli et al., 2016; Branchefsky and Park, 2018; Ollrogge et al., 2022). First, they were asked to indicate their career stage, resulting in the four dummy variables *Student, PhD student, Post-doc*, and *Professor*. It is important to mention that both undergraduate and postgraduate students are included in the first variable. In contrast, the variable *Professor* includes assistant, associate, and full professors. Furthermore, respondents indicated their gender, resulting in the dummy variable *Woman*. Respondents were asked if they have a woman or man professor in the social sports sciences as a role model. If yes, respondents were further asked to indicate the number of women and men role models. Based on this question, the two dummy variables *Woman_Prof_RM* and *Man_Prof_RM* were created, reflecting if the respondent has at least one woman or man professor role model. Respondents were asked in which country they currently study or work at a university. In total, respondents indicated 25 different countries, but only six countries were represented in the sample by more than 1% (*Germany, US, Canada, Australia, Austria, UK*). All other countries are included in the dummy variable *Other_Country*. Finally, respondents' science attitude index was measured with three items based on Young et al. (2013). The index was included as a control variable because gender stereotypes in academic disciplines were found to be related to women's and men's career objectives and interest in the discipline (e.g., Makarova et al., 2019). The items asked for respondents' interest in science and how much they enjoy doing science on a 5-point scale. The science attitude scale can be considered reliable with a Cronbach's alpha of 0.756.

### 3.3 Data analysis

The data were analyzed using IBM SPSS Statistics 28.0 and Stata/MP 17. Descriptive statistics are presented in order to give an overview of the sample structure. Afterward, the role fit indices were estimated based on a procedure that has been used and described by Hallmann and Breuer (2010). They estimated the fit between sport events and the destination image of host cities. A similar procedure has been applied to the context of sport marketing by Musante et al. (1999). The role fit indices (RFI) are based on the Euclidian distance and were estimated according to the following equitation:


$$\begin{array}{l} RFI\;\left(x_{i},y_{i}\right)=1-\sqrt{\overset{n}{\underset{i=1}{\sum }}{\left(x_{i}-y_{i}\right)}^{2}} \end{array}$$

In the following, the procedure is explained in detail using the example of the women's role fit index. For the total role fit index of women academics, all item values of each participant for the women academics attribute scale (*y*_*i*_) were subtracted from the respective item values of the ideal-typical academics scale *(x*_*i*_). For example, if a respondent strongly agrees (5) that academics in the social sports sciences should be authoritarian and disagrees (2) that women academics in the social sports sciences are authoritarian, the result of this first step is 3 for the first item. Similarly, the difference is calculated for all other items. Afterward, the Euclidean distance was calculated. Therefore, the results for all items were added and then divided by the highest squared distance, which is possible. For a 5-point Likert scale, the maximum difference per item is 4, resulting in 256 as the highest possible squared distance for 16 items ([4^*^4]^*^16). This step is necessary to adjust the values to a range between 0 and 1 (Hallmann and Breuer, 2010). After taking the square root, the value was subtracted from 1. The result of this step was the fit index for women academics, with 0 indicating a poor role fit and 1 indicating a perfect role fit. The men's role fit index was created accordingly by using the values from the men academics attributes scale.

Furthermore, the role fit indices for the four dimensions for both women and men academics were calculated by only subtracting the respective items of a dimension and calculating the Euclidean distance as explained above. The sum from the first step was divided by 64 instead of 256 because the maximum possible squared distance for four items is (4^*^4)^*^4. Furthermore, the absolute differences between the perceived men's role fit index and the women's role fit index were calculated (*Fit Diff_Total*).

In the third step, two sets of regression analyses were conducted to investigate the relationship between the perceived role fit indices and individual characteristics. In the first set of models, the total fit indices of women and men academics were used as the dependent variable and the absolute difference between the total fit indices of women and men in an additional model. In the second set, the fit indices for the four different dimensions serve as dependent variables. All independent variables were tested for multicollinearity by estimating correlation coefficients and variance inflation factors (VIF). However, multicollinearity was not an issue in this study. All individual variables displayed in Table 1 were included as independent variables. While a linear regression (ordinary least squares [OLS]) was estimated for the model with the absolute difference between total fit indices as the dependent variable, all other models are fractional response models. Fractional response models are appropriate when the dependent variable is continuous but bounded between 0 and 1, representing a violation of key assumptions of OLS regressions (Papke and Wooldrige, 1996). A significance level of α = 0.05 was applied to all models, and all models were estimated with heteroscedasticity robust standard errors.

## 4 Results

Table 1 shows the descriptive statistics for all role fit indices and individual characteristics of respondents. Starting with the total role fit index, women academics are perceived as having a higher role fit than men academics (0.77 and 0.75, respectively). While the maximum value of 1 (i.e., perfect role fit) is achieved for both genders, women have a lower minimum value of 0.18 than 0.33 for men academics. For the role fit dimensions, women academics achieve a leadership role fit of 0.79 (0.12–1), while men academics' leadership role fit is lower with 0.72 (0–1). The role fit indices are nearly identical for research methods, with an average role fit of 0.81 for women academics and 0.82 for men academics. For research topics, women's role fit is 0.82, ranging from 0.13 to 1. The fit index for research topics for men academics is 0.81, with a smaller range from 0.25 to 1. For media visibility, men academics have only a slightly better role fit than women in this dimension (0.76 for women academics and 0.77 for men academics).

The results of the regression analyses are displayed in Table 3 (for the total role fit indices and the absolute difference between the total fit indices) and Table 4 (for the role fit indices by dimension), with all models being statistically significant. In the following, the results are presented in the order of the developed hypotheses. Starting with the disciplines of the social sports sciences, studying or working in sport management has a significant negative effect on the total role fit index of both women and men academics (Table 3, Model 1a; 1b). Furthermore, being in the discipline of sport management significantly reduces the perceived role fit for the leadership dimension for both women and men academics (Table 4, Model 3a; 3b) and also has a significant negative effect on the role fit of men academics in the media visibility dimension (Table 4, Model 6b). In contrast, studying or working in sports economics significantly increases the perceived role fit of men academics in the leadership dimension (Table 4, Model 3b). Thus, hypothesis 1a is not supported by the results, while 1b can be confirmed for sport economics and leadership fit.

**Table 3:** Fractional response regression models (1a−1b) for the total role fit index and linear regression model (2) for the total fit difference between women and men academics (*n* = 792).

	**1a: Fit W_Total**	**1b: Fit M_Total**	**2: Fit Diff_Total**
Economics	0.007	0.014	0.006
Management	−0.022^*^	−0.024^**^	−0.002
Sociology	0.009	0.002	−0.007
Student	REF	REF	REF
PhD student	−0.036^**^	−0.043^**^	−0.007
Post-doc	−0.050^**^	−0.079^***^	−0.031
Professor	−0.078^***^	−0.085^***^	−0.007
Woman	7.310	−0.028^**^	−0.028^***^
Woman_Prof_RM	0.025	−0.015	−0.042^**^
Man_Prof_RM	−0.021	0.036^*^	0.059^***^
Germany	REF	REF	REF
USA	0.027	0.006	−0.022
Canada	0.028^*^	−0.013	−0.041^**^
Australia	0.006	−0.005	−0.012
Austria	−0.001	−0.010	−0.008
UK	0.031	−0.008	−0.041
Other_Country	0.055^*^	0.035	−0.021
Science attitude	0.006	0.005	−0.008
(*Pseudo*)*R*^2^	0.004	0.007	0.073
χ^2^*/F*	54.65^***^	101.87^***^	3.22^***^

Displayed are the average marginal effects.

* p < 0.05.

** p < 0.01.

*** p < 0.001.

All models estimated with heteroscedasticity robust standard errors.

**Table 4:** Fractional response regression models for the role fit indices of women and men academics by dimension (*n* = 792).

	**3a: Fit W_Leader**	**3b: Fit M_Leader**	**4a: Fit W_Methods**	**4b: Fit M_Methods**	**5a: Fit W_Research**	**5b: Fit M_Research**	**6a: Fit W_Media**	**6b: Fit M_Media**
Economics	0.023	0.038^**^	0.008	0.018	0.014	0.006	−0.006	−0.002
Management	−0.028^*^	−0.033^*^	−0.019	−0.012	−0.012	−0.010	−0.023	−0.038^**^
Sociology	0.022	0.014	0.001	−0.006	0.014	−0.003	0.004	0.006
Student	REF	REF	REF	REF	REF	REF	REF	REF
PhD student	−0.038^*^	−0.053^*^	−0.028	−0.022	−0.044^*^	−0.075^***^	−0.036^*^	−0.027
Post-doc	−0.042	−0.146^***^	−0.058^*^	−0.038	−0.024	−0.088^***^	−0.039	−0.036
Professor	−0.095^***^	−0.143^***^	−0.064^*^	−0.058^**^	−0.065^**^	−0.066^**^	−0.069^**^	−0.055^**^
Woman	0.002	−0.055^***^	0.001	−0.009	0.010	−0.033^**^	−0.005	−0.012
Woman_Prof_RM	0.038^*^	−0.041	0.014	−0.015	0.002	0.009	0.028	−0.005
Man_Prof_RM	−0.008	0.061^*^	−0.009	0.048^*^	−0.009	0.008	−0.031	0.029
Germany	REF	REF	REF	REF	REF	REF	REF	REF
USA	0.035	−0.013	0.010	0.001	0.041^*^	0.040^*^	0.030	0.021
Canada	0.041	−0.030	0.008	−0.019	0.057^**^	0.009	0.008	−0.002
Australia	0.005	−0.001	−0.041	−0.042	0.037	0.058^*^	0.007	−0.018
Austria	0.029	−0.020	−0.010	−0.016	−0.001	0.035	−0.042	−0.040
UK	0.024	−0.041	0.002	0.007	−0.007	−0.014	0.105^**^	0.032
Other_Country	0.092^**^	0.063	0.039	0.029	0.040	0.048	0.046	0.014
Science attitude	0.009	0.009	0.012	0.006	−0.001	0.005	0.005	0.001
*PseudoR* ^2^	0.009	0.027	0.004	0.006	0.006	0.007	0.004	0.004
χ^2^	50.67^***^	133.15^***^	26.81^*^	34.82^**^	33.59^**^	53.18^***^	32.53^**^	33.54^**^

Displayed are the average marginal effects.

* p < 0.05.

** p < 0.01.

*** p < 0.001.

All models estimated with heteroscedasticity robust standard errors.

For the career stages, the group of students serve as the reference category. There are significant negative effects on the total role fit index of both women and men academics for PhD students, Post-docs, and professors (Table 3, Model 1a; 1b). Similarly, all three groups have a significantly lower likelihood of perceiving a high role fit for men academics in the dimensions of leadership and research topics (Table 4, Model 3b; 5b). For women academics, PhD students and professors are more likely to perceive a lower role fit in the dimensions of leadership, research topics, and media visibility compared to students (Table 4, Model 3a; 5a; 6a), while a significant negative effect can be found for post-doc researchers and professors and their perception of women academics' role fit in the dimension of research methods (Table 4, Model 4a). For the fit indices of men academics in the dimensions of research methods and media visibility, professors are more likely to perceive a lower role fit than students (Table 4, Model 4b; 6b). Therefore, hypothesis 2b must be rejected, while hypothesis 2a can be confirmed.

Women respondents perceive a significantly lower total role fit index for men academics (Table 3, Model 1b). Furthermore, the absolute difference between men's and women's role fit index is significantly lower for women (Table 3, Model 2). For the dimensions, woman's gender significantly reduces the perceived leadership role fit of men academics and the fit of men academics in the dimension of research methods (Table 4, Model 3b; 4b). Men were found to perceive a higher role fit for men academics, while women perceived a lower role fit for men academics and a lower difference between women's and men's role fit (Table 3, Model 1b; 2). Hypothesis 3a can be confirmed for the absolute difference in the perception of women's and men's role fit. However, no significant effects are evident on women academics. Furthermore, hypothesis 3b is fully confirmed by the results.

The absolute fit difference between women and men academics is significantly lower for respondents with a woman role model (Table 3, Model 2). Furthermore, having a woman role model increases the perceived role fit of women academics in the leadership dimension (Table 4, Model 3a). Similarly, having a man role model has a significant positive effect on the total role fit of men academics and the absolute difference between women's and men's role fit (Table 3, Model 1b; 2). Furthermore, having a man role model has a significant positive effect on the role fit of men academics in the dimensions of leadership and research methods (Table 4, Model 3b; 4b). These results support the hypotheses 4a and 4b.

For the different countries included in the analysis, several variables show significant results. Compared to respondents from Germany, respondents from Canada are significantly more likely to perceive a higher total role fit of women academics and perceive a lower total difference between women's and men's role fit (Table 3, Model 1a; 2). Furthermore, a significant positive effect can be found for men's role fit in the dimension of research methods for people who work or study in Canada (Table 4, Model 4b). Working or studying in the US increases the perceived role fit index in the dimension of research topics for both women and men academics (Table 4, Model 5a; 5b). No significant effects can be found for respondents from Austria. However, hypothesis 5a can be confirmed for the higher perceived role fit of women academics by respondents from Canada and the US. Hypothesis 5b is not supported by the results of the study. Furthermore, respondents from other countries perceive a significantly higher total role fit for women academics and a higher role fit in the leadership dimension (Table 3, Model 1a; Table 4, Model 3a).

## 5 Discussion

This study examined the role fit of women and men academics and which individual characteristics are related to the perception of role fit and the difference in role fit. The total role fit of women academics is higher than the perceived role fit of men academics, indicating that women are not perceived as a mismatch for academic positions in the social sports sciences. This result is contradictory to the assumed lack-of-fit (Heilman, 2012) and role congruity theory (Eagly, 1987; Eagly et al., 2000). Women academics in the social sports sciences seem to be accepted, maybe because they work in a men-dominated discipline and are associated with normally stereotypical men attributes (Sendén et al., 2019). Furthermore, women and men academics appear to converge regarding the perception of their role attributes. The finding for the total role fit index is in line with Renström et al. (2021), who found that women lecturers are not perceived differently from men lecturers by students. Furthermore, it supports the findings of Bye et al. (2022) that women academics are not only perceived as similar to men academics but are also rated higher in some attributes. Even though the total role fit is higher for women academics, the range of the role fit indicates that women might be perceived more controversially than men. The minimum role fit for women academics is much lower than the value for men academics. Therefore, it is possible that some people in the social sports sciences still perceive that “women do not have what it takes” (van Veelen and Derks, 2022b, p.750). However, these people seem to be a minority.

The role fits of the four dimensions provide a deeper insight into the perception of women and men academics in the social sports sciences. Starting with the leadership dimension, women are perceived as a better fit to the role of a leader than men academics. This finding is surprising, since jobs which require leadership abilities were found to violate the social role of a woman (Prentice and Carranza, 2002). The higher role fit for women academics supports the finding that the perceived role incongruity between women's social role and the role of a leader becomes smaller and that women leaders are evaluated more positively than men leaders (Tremmel and Wahl, 2023). Even though women are minorities in leadership positions in the social sports sciences (e.g., Federal Statistical Office, 2022), the result for the leadership dimension indicates that women are perceived as better leaders than men. One explanation for this result might be that women leaders in men-dominated disciplines combine both typically feminine and typically masculine attributes. This finding is in line with a study from the corporate sector, where women leaders' stereotypes did not differ from those of men leaders (Eriksson et al., 2021). Further research is needed to investigate whether communal attributes, which are associated with the social role of women, are generally more valued in the social sports sciences than agentic attributes, which are related to men's social role (Eagly and Karau, 2002; Prentice and Carranza, 2002). However, it might also be possible that women academics in the social sports sciences are rated higher on stereotypical men agentic attributes than men.

The differences between women's role fit and men's role fit for the other three dimensions are marginal (0.01), again indicating that both genders seem to be equally matching the role of an academic. With a role fit of over 0.80 for the dimensions of research methods and research topics, both women and men academics appear to be rated competent with respect to science- and discipline-specific topics and skills. These findings are contradictory to previous research suggesting that women are perceived as having less analytical skills (Calanca et al., 2019) and do not fit quantitative research (Bettinger and Long, 2005). The nearly identical fit indices for women's and men's media visibility (0.76 for women and 0.77 for men, respectively) are surprising, given that women are less visible than men in academic journals in all three disciplines of the social sports sciences (Pitts et al., 2014; Gomez-Gonzalez et al., 2022; Wicker et al., 2022). Participants in this study were people who work or study in the social sports sciences. The community of the social sports sciences is comparably small, meaning that many people know each other from academic conferences or collaborations. Therefore, it is possible that these people are more likely to consciously follow their women and men colleagues and/or connect online.

Turning to individual characteristics which are related to the perception of women's and men's role fit, the discipline seems to be relevant. As hypothesized, respondents in sports economics perceived a higher role fit for men academics, even though the effect is just significant for the leadership dimension. In sports economics, men are perceived as better leaders, indicating that academics in this discipline have a more traditional view of leadership and the social role and related qualities and behaviors of men (Eagly, 1987). Therefore, it might be especially important to actively promote women in sports economics. With an increasing share of women in the field, gender stereotypes could start to decrease, resulting in a more gender-equal perception of leadership role fit. Interestingly, academics who study or work in sport management perceive a lower total role fit for both women and men academics, as well as a lower leadership role fit for both genders. This result might be explained by the fact that leadership is an important research topic within sport management. Academics in sport management do research on leadership styles, gender and leadership, behavior and effectiveness of leaders, as well as outcomes and ethical aspects (Welty Peachey et al., 2015). Through their professional knowledge of desirable attributes and behaviors of a leader, it is possible that people within sport management evaluate academics in the leadership dimension more critically. This explanation seems to be logical, given that the leadership role fit both genders is negatively related to sport management. Furthermore, this evaluation could also be the reason for the effects in the models with the total role fit.

Academics in the earliest career stages, i.e., undergraduate and postgraduate students, perceive a higher role fit than academics in later career stages. However, the perception is not related to one gender. Again, these findings again support the image that role incongruity (Eagly and Karau, 2002) between women's social role and women's role in academia is not perceived in the social sports sciences. While van Veelen and Derks (2022b) found that both women and men professors perceive a high fit between themselves and their academic role, the results of this study indicate that people at later career stages are more critical toward the role fit of others in the social sports sciences. While the effects for PhD students and post-doc researchers are not significant in all models, professors in the social sports sciences perceive a lower role fit for the total fit but also for all dimensions than students. It is possible that professors know more about “what it takes” to fit the role of ideal-typical academic, resulting in higher expectations. Qualitative research could help to gain a deeper understanding of how people at earlier and later career stages evaluate others within their discipline.

Respondents' gender seems to be related to the perception of men academics' role fit. Women perceive a smaller difference between the role fit of women and men. In contrast, men perceive a higher role fit for men academics. Even though the regression models with women's role fit as a dependent variable do not show significant effects for gender, the effects in the other models support findings that men perceive a stronger role incongruity between women and the work in academia (Carli et al., 2016), while women perceive a higher role fit for women academics (Bye et al., 2022). The results support previous literature that men perceive stronger gender-science stereotypes (Symth and Nosek, 2015), resulting in the perception of a higher role fit for men academics. The smaller difference in the perceived role fit by women is in line with Diekman et al. (2004), who found that women perceive the ongoing change in traditional gender roles more positively than men. In a broader societal context and in line with previous studies (e.g., Villanueva-Blasco and Grau-Alberola, 2019), the results indicate that women have internalized the traditional gender roles to a smaller extent and are less likely to accept these socially constructed roles than men.

While having a women professor in the social sports sciences as a role model is not related to the total role fit, it leads to a smaller difference between the perception of men's and women's role fit and a higher leadership fit. Contrary, having a men role model increases the difference. It is related to higher leadership and research methods fit for men academics. Again, these results suggest that men tend to have stronger gender-science stereotypes (Symth and Nosek, 2015). The results indicate that women role models in academia are not only relevant for other women but also for men within the field. This result adds to previous findings that women role models are especially important for women (Lockwood, 2006) and that they have a positive impact on women's gender stereotypes (Olsson and Martiny, 2018). Having a women professor as a role model implies the recognition of the women role models' achievements, skills, and competencies. Accordingly, people with a women role model perceive less role incongruity than those with a men role model. In line with role congruity theory (Eagly and Karau, 2002), academics with a men role model seem to evaluate academics through the lenses of traditional, social gender roles and attribute men's characteristics more to the role of a successful academic. Overall, the findings indicate that women role models are important to reduce gender stereotypes in the social sports sciences, and more women in higher academic positions (e.g., professors) are needed.

Academics who study or work in Canada are more likely to perceive a higher total role fit for women academics, a smaller difference between women's and men's total role fit, and a higher fit for women in the leadership dimension (compared to academics in Germany). Even though Germany is ranked better in the total gender gap index, Canada has a smaller gender gap in economic participation and education (World Economic Forum, 2022). Both dimensions are directly related to studying or working in academia. Thus, the results might be explained by the smaller gender gap in academia in Canada, in line with Mòe et al. (2021). From a theoretical perspective, these findings suggest that academics in Canada perceive a higher role congruity (Eagly and Karau, 2002) for women academics. Interestingly, respondents from other countries than those included as separate variables in the analysis perceived a higher total role fit for women and women leaders than academics from Germany. These findings need further investigation because the variable includes more than 20 different countries, each of them represented by < 1% of the respondents. However, the result suggests that gender stereotypes in academia seem to be stronger in Germany than in several other countries in the world. In Germany, the Conservative party has won the federal election between 2005 and 2021 (German Parliament, 2021). The party advocates traditional social roles of women and men (Geissel, 2013). According to previous literature, conservative political ideologies were found to be related to gender stereotypes (Hoyt, 2012).

Overall, the results of this study suggest that women's and men's academics in the social sports sciences both fit the role of an academic. Surprisingly, women achieved a higher total role fit and a higher fit in the leadership dimension, indicating that role incongruity (Eagly and Karau) is less present in the disciplines of the social sports sciences or that they have started to decrease. Furthermore, individual characteristics, such as the career stage, gender, and the country in which one studies or works, were found to be related to the perception of the role fit and the difference between women's and men's role fit.

## 6 Conclusion

Based on social role theory (Eagly, 1987; Eagly et al., 2000) and role congruity theory (Eagly and Karau, 2002), the aim of this study was to investigate the role fit of women and men academics in the social sports sciences and how individual characteristics are related to the perception of role fit as well as the difference in the perception of women and men academics. In the social sports sciences, women are perceived as a better fit for the role of an academic than men. These findings question the current gender representation in the social sports sciences since women are still underrepresented in sport faculties. The underrepresentation is especially evident in later career stages. Given that women's academics are perceived as a better fit for the leadership dimension, the share of women in those top positions should be increased. Actions to increase the share of women in academia might help to further break down gender stereotypes and traditional role attributions. The latter is especially important because men still seem to perceive a stronger role congruity between men and men academics.

The results of this study can contribute to the body of literature in several ways: firstly, this study investigates gender stereotypes by measuring the perception of stereotypically masculine and feminine attributes in the social sports sciences, a field which is associated with masculinity (Burton, 2015), but which also includes research fields which are considered more typical for women (Su et al., 2009; Conde-Ruiz et al., 2022). Previous studies were mostly focused on STEM disciplines, while lacking the social sciences (Johnson et al., 2022). Secondly, previous studies were mostly focused on the effect of gender stereotypes on women, e.g., for their career path (Olsson and Martiny, 2018), while this study investigates the role fit of both women and men academics. Thirdly, the present research is the first study estimating role fit indices for women and men in academia. The role fit indices were also presented for four dimensions, revealing a more nuanced look at how academics perceive other academics within the social sports sciences. Fourthly, several individual characteristics, which have been found to be related to the perception of gender stereotypes, were included to understand how they are associated with the perceived role fit.

The study provides some implications for both researchers in the field of gender stereotypes in academia and researchers who study or work in the social sports sciences. The study shows the importance of investigating gender stereotypes and role (in)congruities in different disciplines. It is not possible to generalize findings from other disciplines, even if many circumstances seem comparable. Practically, academics in the social sports sciences need to reflect their own stereotypes, especially when they are involved in performance evaluations or hiring decisions. Workshops within the faculties or at conferences might be a good start. It may be advantageous to increase and encourage communication between the disciplines of the social sports sciences, e.g., by organizing a joined conference, since academics in different disciplines perceive different role fits. Since the majority of academics within the field are men and the results suggest that men tend to perceive a better fit of men academics, it is especially important that men are actively encouraged to reconsider their view on role fit. Furthermore, the regression results indicate that women's role models may be helpful because they showcase that women's social role has developed and that they have had the skillset to become a full professor. While previous studies found that women role models are especially important for women (Lockwood, 2006), they can also shape men's perception of role fit.

The study has some limitations, which can guide future studies. The dataset reflects cross-sectional data and is limited to the time of data collection. Longitudinal studies would enrich the state of knowledge by seeing if and how the role fit changes during people's academic career. The online survey was designed to measure the perception of role fit, not the actual behaviors directed toward gender. A mixed-methods approach might be valuable to get a deeper understanding of a potential bias between the perception and behaviors. Furthermore, the items in the role attributes scale cannot measure all aspects that might be important in the four dimensions. Given that this study is the first that estimates a role fit index and that multi-dimensional approaches were described as beneficial (Haines et al., 2019), future studies could include more items for each dimension. Participants of this study were people who study or work in the social sports sciences, and they were asked to participate in the study. Therefore, a selection bias might be present, meaning that people who are generally more open to the topic of gender stereotypes, who perform research on the topic or a related topic like gender diversity, or who support efforts to increase the share of women in the social sports sciences, might have participated in the study while others were not interested. The sample only reflects respondents who identify as women or men. Future studies could expand the investigation of role fit beyond the binary gender considerations. In addition, previous studies suggest that individuals' gender attributes are perceived differently depending on their sexual orientation (Shamloo et al., 2022; Salvati et al., 2023). Therefore, investigating intersectionality in the context of gender stereotypes and the perception of role fit in academia would be an important extension of current research. Finally, respondents to this study were people who work or study in the social sports sciences. It might be possible that people who are already within the academic system and a certain discipline generally have a higher perception of role fit of both genders and that people with a lower perceived role fit in the general population never enter an academic career in the social sports sciences.

## Data availability statement

The raw data supporting the conclusions of this article will be made available by the authors, without undue reservation.

## Ethics statement

The studies involving humans were approved by Ethics Commitee of Bielefeld University. The studies were conducted in accordance with the local legislation and institutional requirements. The participants provided their written informed consent to participate in this study.

## Author contributions

LL performed the data cleaning and data analysis under the advice of PW, drafted the introduction, theory and literature review, method, results, discussion, and conclusion. KS and PW checked the overall manuscript for coherence, language, and format. All authors were part of the conceptualization of the study and the design of the questionnaire, contributed to the data collection, revised the manuscript critically, and agreed on the final version of the manuscript.
